# A Comparative Molecular Dynamics Study of Selected Point Mutations in the Shwachman–Bodian–Diamond Syndrome Protein SBDS

**DOI:** 10.3390/ijms23147938

**Published:** 2022-07-19

**Authors:** Elena Spinetti, Pietro Delre, Michele Saviano, Dritan Siliqi, Gianluca Lattanzi, Giuseppe Felice Mangiatordi

**Affiliations:** 1 Department of Physics, Trento Institute for Fundamental Physics and Applications, Frankfurt Institute for Advanced Studies, Ruth-Moufang-Straße 1, 60438 Frankfurt am Main, Germany; spinetti@fias.uni-frankfurt.de; 2 Institute of Crystallography, National Research Council of Italy, Via Amendola, 122/o, 70126 Bari, Italy; pietro.delre@ic.cnr.it (P.D.); dritan.siliqi@ic.cnr.it (D.S.); 3 Institute of Crystallography, National Research Council of Italy, Via Vivaldi, 43, 81100 Caserta, Italy; michele.saviano@cnr.it

**Keywords:** Molecular Dynamics, Shwachman–Diamond Syndrome, Shwachman–Bodian–Diamond Syndrome protein

## Abstract

The Shwachman–Diamond Syndrome (SDS) is an autosomal recessive disease whose majority of patients display mutations in a ribosome assembly protein named Shwachman–Bodian–Diamond Syndrome protein (SBDS). A specific therapy for treating this rare disease is missing, due to the lack of knowledge of the molecular mechanisms responsible for its pathogenesis. Starting from the observation that SBDS single-point mutations, localized in different domains of the proteins, are responsible for an SDS phenotype, we carried out the first comparative Molecular Dynamics simulations on three SBDS mutants, namely R19Q, R126T and I212T. The obtained 450-ns long trajectories were compared with those returned by both the open and closed forms of wild type SBDS and strongly indicated that two distinct conformations (open and closed) are both necessary for the proper SBDS function, in full agreement with recent experimental observations. Our study supports the hypothesis that the SBDS function is governed by an allosteric mechanism involving domains I and III and provides new insights into SDS pathogenesis, thus offering a possible starting point for a specific therapeutic option.

## 1. Introduction

Shwachman–Diamond Syndrome (SDS, OMIM #260400 and #617941) is an autosomal recessive disease that affects many parts of the body, including bone marrow, pancreas, bones, immune and the central nervous system [1], with an increased risk of progression to myelodysplastic syndrome [2]. In most cases, SDS is associated with mutations in the Shwachman–Bodian–Diamond Syndrome gene (SBDS, OMIM gene #607444), located on chromosome 7q11 and encoding for a protein, named SBDS, structurally organized into three highly conserved domains [2,3,4]. SBDS is required for the assembly of mature ribosomes and ribosome genesis. Together with Elongation Factor Like-1 EFL1 (OMIM gene #617538), SBDS triggers the GTP-dependent release of eIF6 (eukaryotic initiation factor 6) from 60S pre-ribosomes in the cytoplasm [2,5,6], thereby activating the ribosomes for translation competence by allowing 80S ribosome assembly and by facilitating eIF6 recycling to the nucleus, where it is required for 60S rRNA processing and nuclear export. In this mechanism, SBDS acts as the nucleotide exchange factor (GEF) for EFL1 [7,8,9], increasing its affinity for GTP over GDP [5]. Most SDS patients display the biallelic pathogenic variants c.183_184TA > CT (K62X) and c.258 + 2T > C (C84fsX3) [10]. Less common mutations have also been reported throughout the gene including nonsense mutations, missense mutations, small deletions, indel conversions and splice-site mutations [10,11,12,13,14]. The rest of the patients (less than 10%) present clinical indications of SDS without having any pathogenic variants in SBDS but in other genes coding the proteins DNAJC21 [15,16], SRP54 [17] and EFL1 [18,19,20,21]. Importantly, the observed SBDS mutations result in a protein function drop that is responsible, when the SBDS levels are below a certain threshold, for a phenotype corresponding to the clinical manifestation of SDS. SBDS missense mutations can be grouped into those affecting the stability of the protein and those that modify surface epitopes without altering the protein fold [2]. Unfolding of SBDS results in limited amounts of protein available to fulfill its function, but the impact that surface modifications may have on the function of SBDS is still largely unknown. Due to the GEF role of SBDS for EFL1 [5], it would be expected that decreased amounts of SBDS may not be sufficient to activate EFL1 at the levels necessary to elicit the required physiological effect. The first report of severe SDS syndrome phenotype [22] caused by compound heterozygous missense mutations in SBDS, N121T/R175W, consisting of a stability and a surface mutation, respectively, suggests that the presence of an insufficient protein quantity is detrimental not only for the activity of SBDS but also for ligand recognition through its surface epitope. This indicates that the missense mutations clinically manifesting an SDS phenotype may alter the protein conformational plasticity, which is probably crucial for its function. In our previous paper [23] we reported seven surface missense mutations, thus investigating the EFL1-SBDS interaction by applying a model with two different binding sites. Both proteins (in their wild type and mutant isoforms) were expressed according to their yeast sequences and analyzed via fluorescence anisotropy experiments. This study pointed out that all three SBDS domains are involved in the specific interaction with EFL1, with domain 1 and domains 2–3 acting as independent entities. More precisely, domain 1 constitutes the region undergoing the conformational changes, and its interaction with EFL1 becomes avid by a “chelate” effect with domains 2–3 of the protein. These conformational changes of SBDS were further supported by SAXS experiments performed on this protein in solution and not bound to the 60S ribosome subunit. Aimed at getting unprecedented insights into the molecular mechanism underpinning SBDS function/dysfunction, we carried out comparative Molecular Dynamics (MD) investigations on a panel of SBDS mutants, namely R19Q, R126T and I212T. In a previous co-authored paper [23], we showed that the R19Q mutant was responsible for a decrease in the affinity of the second binding event; the R126T mutant displayed a decreased affinity for both binding events; finally, the I212T mutant severely disrupted EFL1 binding. These mutations were selected for the following reasons: (i) they are localized in different domains (domain I, II and III, respectively); (ii) were proved to induce the same effect, namely an altered SBDS-EFL1 binding; (iii) none of them lies in close proximity to EFL1 in the cryo-EM structures when bound to the 60S subunit [5]. In other words, they represent ideal case studies to properly investigate, by all-atoms MD simulations, the complex conformational landscape governing the SBDS function and, therefore, SDS pathogenesis.

## 2. Results

### 2.1. Wild Type SBDS Is More Stable in the Closed Conformation

**Figure 1:**
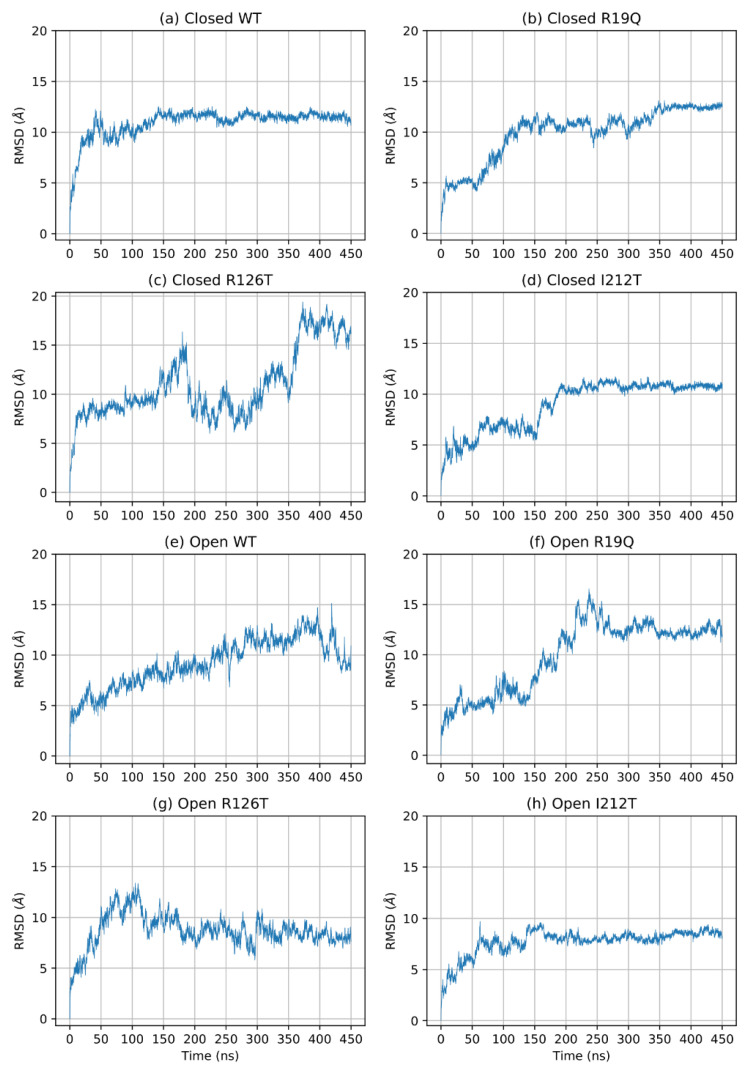
*RMSD* of Cα of the entire protein during simulation.

To understand the effects of mutations on the structure of SBDS, we performed an in-depth analysis of the MD trajectories obtained from the simulations of eight molecular systems, namely the wild type (WT) form of SBDS obtained from both the closed and open NMR structures and the mutants R19Q, R126T and I212T. As a first step, we investigated the time-dependence of the root mean square deviations (*RMSD*) computed for all the alpha carbon atoms (Cα). Different structures required different simulation times to equilibrate as can be seen in Figure 1. Remarkably, the open WT structure is the only system that displayed a significant drift. The estimated equilibration times are reported in Table 1. Subsequent portions of the trajectories are supposed to sample the equilibrium NPT ensemble.

**Figure 2:**
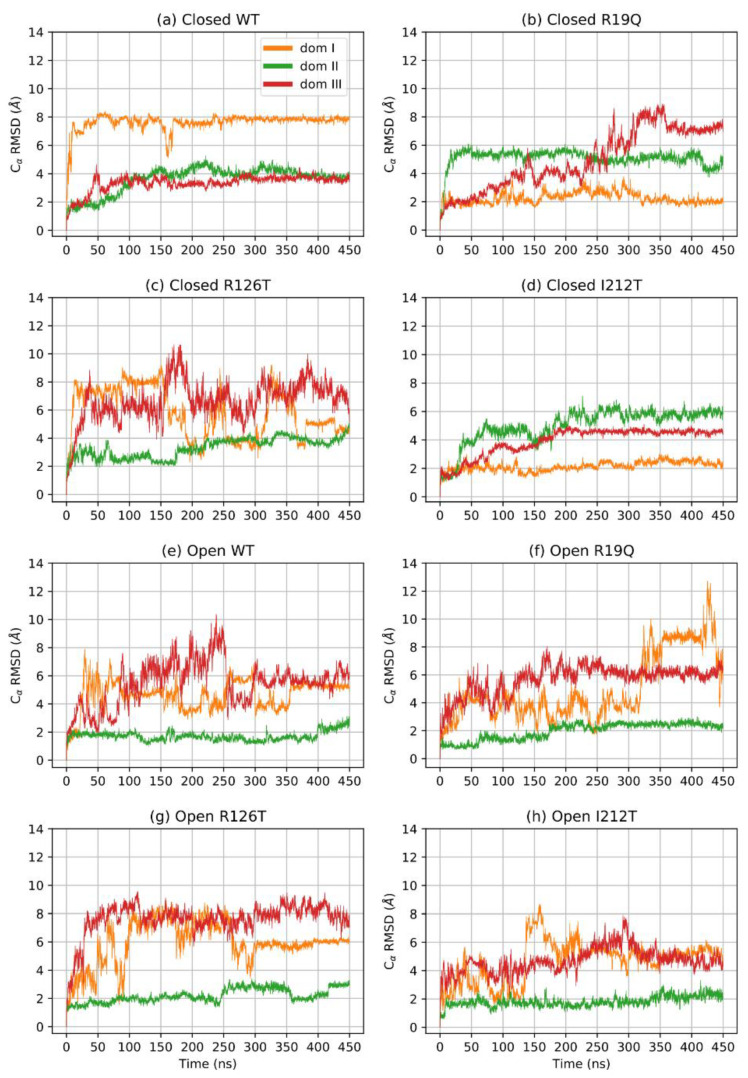
*RMSD* of Cα per domain.

A further assessment of structure equilibration can be obtained by an analysis of the *RMSD* computed for each protein domain. Figure 2 shows the *RMSD* of the three domains for each simulated structure: the closed forms are, in general, more stable than open forms. In particular, the closed WT SBDS equilibrates faster than other mutant structures, although all closed structures can be assumed to be stable after 170 ns. On the contrary, the open WT structure is characterized by a longer equilibration time for all domains: the equilibration point can be estimated to be at least 400 ns. In general, domains I and III are less stable when compared to domain II, even in closed structures. It is interesting to note that the investigated mutations tend to alter the dynamical behavior of the closed conformations, rather than open ones. In addition, a mutation on the domain I (R19Q) seems to affect the dynamics of domain III, rather than domain I itself while a mutation on domain III (R126T) affects both domains I and III.

### 2.2. Closed R126T and Open WT Display the Highest Fluctuations

**Figure 3:**
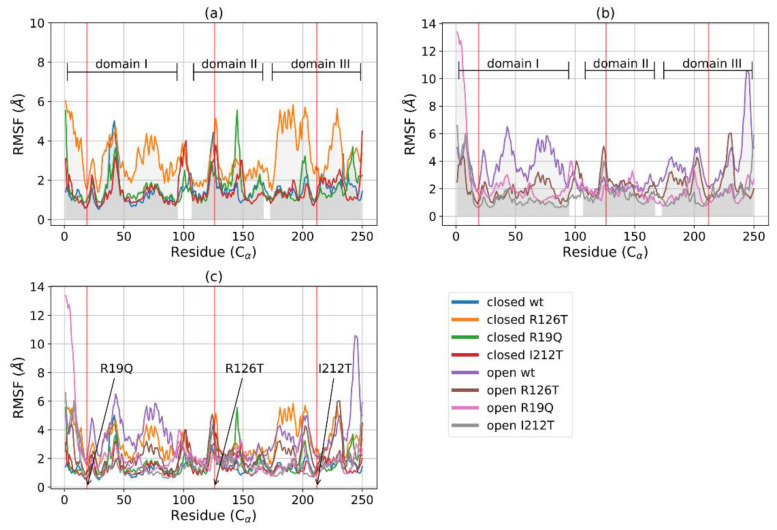
*RMSF* of C for closed (**a**) and open (**b**) structures: WT, R126T, R19Q, I212T. The gray areas indicate the three domains of SBDS: domain I, C1-97; domain II, C 109-170; domain III, C175-252. Red vertical lines point out the mutated residues in position 19 (21 in reference PDB) in domain I, 126 (128 in reference PDB) in domain II and 212 (214 in reference PDB) in domain III. (**c**) *RMSF* for C of all systems (lower horizontal bars indicate different domains).

The root mean square fluctuations (*RMSF*) analysis reveals that, among the closed structures, the mutant R126T displays the highest fluctuations, especially inside domains I and III, where values are much higher than those of the other closed systems (Figure 3a). The other closed mutants show an *RMSF* profile comparable with the wild type. In the case of open systems (Figure 3b), it can be noted that fluctuations are higher for domains I and III in the WT structure with respect to all the other mutated proteins. An overall structure comparison, as reported in Figure 3c, shows that the flexibility of the various structures differs mainly in the Cα of domain I (especially between residues 50 and 100) and domain III (mainly between residues 170 and 220), while they are more similar in domain II. The highest fluctuations are reported for the closed R126T and open WT structures, whose profiles are similar. The effect of the mutation R126T on the overall stability of the protein, even in the closed conformation, is thus confirmed by *RMSF* data. However, the fluctuations decrease in the mutant open conformations with respect to wild type, in particular for domain III. Taken together with the *RMSD* equilibration analysis, the *RMSF* data suggest thus that the instabilities introduced in the structures upon mutations affect mainly the dynamics of domains I and III, while domain II maintains mostly a similar behavior of low fluctuations for all investigated systems. 

### 2.3. Similarity Analysis

Open/closed transitions of the SBDS conformation occur spontaneously in physiological solutions, hence the protein pool should be represented in percentages of closed and open structures [23,24]. Introducing mutations that affect the protein structure stability may alter the population of these conformations. Hence, we introduced the ∆*RMSD* (∆σ) as a progress variable to describe the transition between the closed and the open conformation of SBDS, following ref. [25]. In the present work, ∆*RMSD* was used to quantify the degree of similarity to closed or open reference structures of the mutants.

**Figure 4:**
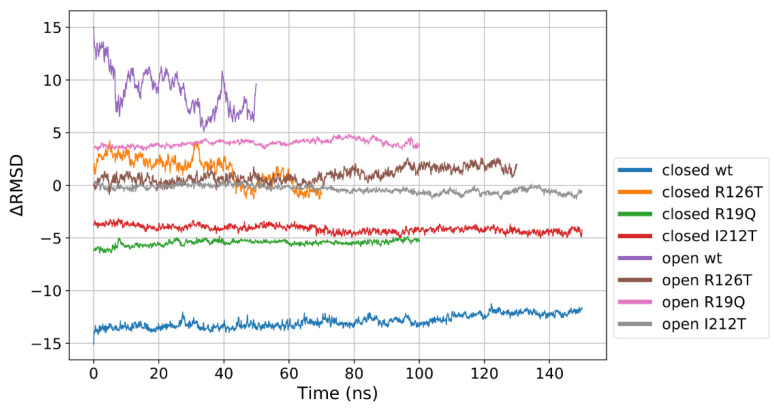
Δ*RMSD* calculated for the different investigated systems. The reference closed and open WT structures mark the extremes of the range of values. The different lengths of the data correspond to the different portions of the trajectories that were considered production runs. The values also indicate that neither the closed nor the open WT trajectories could be considered fully equilibrated, since the former displays a slight drift, while the latter is still fluctuating.

We defined ∆*RMSD* as:

$$\Delta \sigma \left(t\right)=\sigma_{closed}\left(t\right)-\sigma_{open}\left(t\right)$$

where *σ* are the *RMSD* with respect to the reference equilibrated WT closed or open trajectory and are computed as:

$$\sigma^{open}\left(t\right)=\sqrt{\frac{1}{N}\overset{N}{\underset{i=1}{\sum }}{\left(r_{i}\left(t\right)-r_{i}^{open}\right)}^{2}}$$

where $r_{i}\left(t\right)$ are the coordinates of atom *i* at time *t*, *N* is the number of atoms (alpha carbons), $r_{i}^{closed}$ are the coordinates of atom *i* in the first frame of the closed WT production run and $r_{i}^{open}$ are the coordinates of atom *i* in the first frame of the open WT production run. With this approach, we obtained a value of ∆*RMSD* ranging from −15 to +15: a negative value implies that the structure closely resembles the WT closed conformation, while a positive value would denote a close similarity with the WT open conformation. Values close to zero account for intermediate situations. Figure 4 clearly demonstrates that the wild-type systems occupy by definition the extremes of the plot, being the reference for closed and open structures. Most of the proteins display a stable value of ∆*RMSD*, meaning that transitions between the two conformations are not observed within the production runs here examined. The low values for the open I212T conformation qualify this mutant as a candidate for an intermediate form between the closed and open state. An interesting behavior is observed for the case of the closed R126T which displays positive values of ∆*RMSD* in the initial part of the production run but drifts towards the zero value that would correspond to an intermediate state. Again, this clearly indicates that this mutation affects the stability of the closed conformation. However, this analysis suggests that all the investigated mutations tend to produce an intermediate state between the WT open and closed structures.

### 2.4. Analysis of Domain Motions

**Figure 5:**
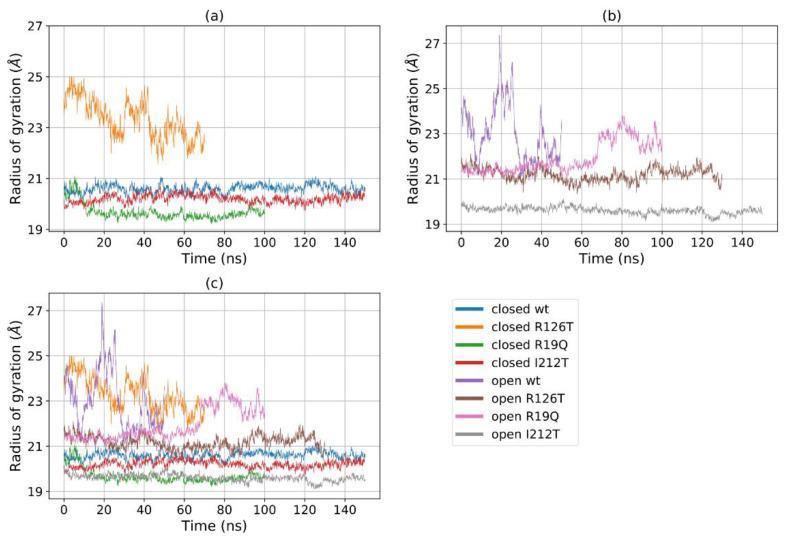
*R_g_* of (**a**) all the eight investigated systems, (**b**) all the closed systems, (**c**) all the open systems.

**Figure 6:**
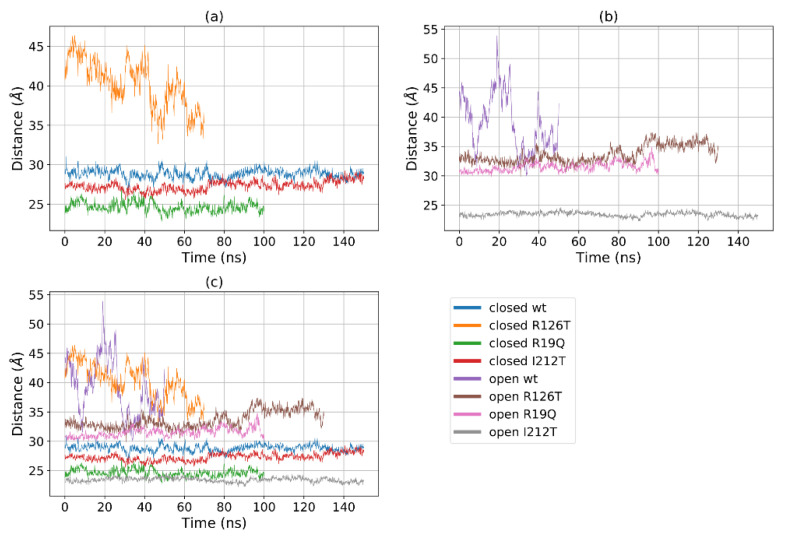
Distances between the centers of mass of domains I and III for (**a**) all the investigated systems, (**b**) closed systems and (**c**) open systems.

Figure 5a shows that for most structures the radius of gyration, at least in the closed states, is stable in the range 19–22 Å, with the notable exception of R126T, whose radius of gyration (*R_g_*) even in the closed state oscillates between 21 and 27 Å. In addition, the open WT conformation displays a high value of *R_g_*, as the open R19Q whose *R_g_* value increases at about 60 ns of the supposed production run. These results are comparable, although to a smaller extent, to the results obtained by Gijsbers et al. [23], who analyzed an ensemble of yeast SBDS conformations that comprised a ‘compact’ conformation with an *R_g_* of 24 Å, a ‘stretched’ one with an *R_g_* of 28 Å and a ‘relaxed’ conformation with an *R_g_* of 30.4 Å. In the present case, the ‘compact’ conformation is analogous to the closed state, while the ‘stretched’ is similar to our open state. These results confirm our previous observation, that some mutations alter the population of the closed/open forms towards an intermediate state. In particular, the closed R126T has characteristics shared by the pool of open systems, while the open I212T, assuming the most compact conformation, may be connected more to a closed state rather than an open state. This tendency is confirmed by the analysis of the mutual domain distances (Figure 6). The mutual distances between domains I and II for the closed R126T are more similar to those of open structures, while the values of the open I212T are quite different from the other open structures and more similar to the values obtained for closed systems (Figure 6a); this difference is better appreciated in Figure 6b,c. This observation adds to previous comments about the similarity of the closed R126T to open structures and the similarity of open I212T to closed structures. A comparison of the closed R126T and the open WT conformations reveals that the mutual distance between domains I and III and *R_g_* are very similar. This may indicate that the *R_g_* variation for these systems can be explained by the relative motion of these two domains. All other structures display stable and lower distances. The open R19Q has a constant value of the distance between domain I and domain III over time, so in this case, the variation in *R_g_* is not related to the distance between domain I and III, but rather to their lower stability.

**Figure 7:**
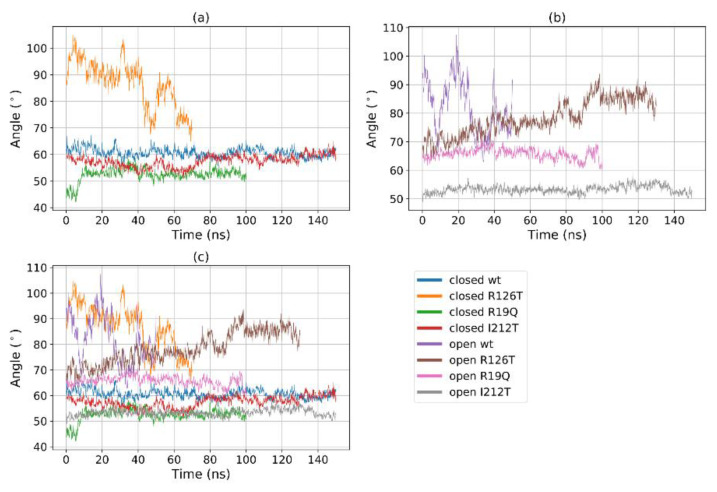
The Angle between the centers of geometries of domains I–II–III over time for (**a**) all the systems; (**b**) closed systems and (**c**) open systems.

A further assessment of domain motions can be obtained by considering the angle formed by the vectors that join the center of geometry of domain II with the centers of the geometry of the other two domains. This angle can be also directly measured: for instance, in yeast SBDS, the ‘compact’ conformation was characterized by an angle of 95°, while the ‘stretched’ one was characterized by an angle of 112° [23]. In our analysis, this angle is stable throughout the trajectory for most structures, with values ranging from 50° to 70° (Figure 7). However, again the closed R126T and open WT single out with a highly fluctuating value that exceeds 100°, consistently with the *R_g_*-based investigation. We remark, however, that the values found in this computational investigation differ from the reported experimental ones, as reported in ref. [23]. In our case, it is not easy to assess the difference between the angles for the closed and open conformations since this value oscillates wildly around 75° for the open WT, while it remains constant at around 60° for the closed WT (Figure 7b). We may therefore conclude that, although the quantitative values differ, the qualitative behavior is consistent with the reported experimental data, and, again the mutant I212T tends to adopt dynamics that closely resemble the closed WT state, while the mutant R126T displays a clear tendency towards the open WT state.

### 2.5. Protein Structure Network Hubs

**Figure 8:**
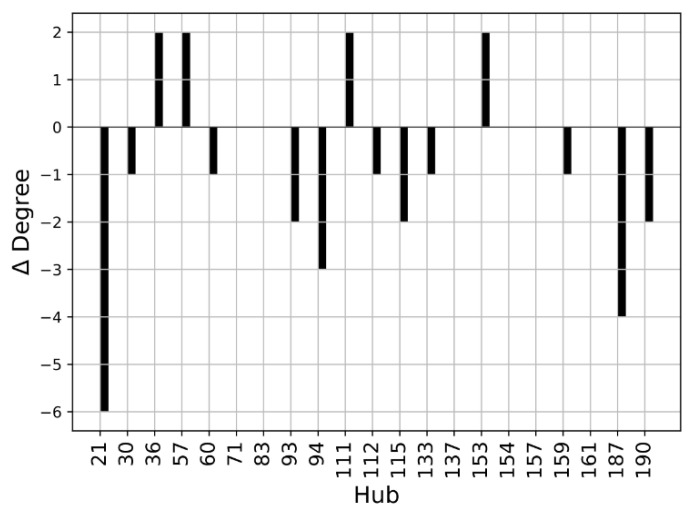
Hub degree comparison of WT structures: Degree (*k_open_* − *k_closed_*) of residues with a connectivity degree higher than 4 (*k* > 4) in at least one of the two proteins. Residues are enumerated according to the PDB notation.

We performed a Protein Structure Network (PSN) analysis of non-covalent interactions to obtain further insight into the investigated structures. The hubs of a protein structure network are significant, as they establish a lot of interactions and can transport information coming from different parts of the protein. The hubs can be seen as fundamental interaction points in the protein structure and a mutation altering one of these hubs may cause dramatic structure rearrangements. As a first step, we compared the hubs of the WT structures. The residues with a connectivity degree higher than 4 (*k* > 4) in at least one of the two wild-type conformations (closed or open) were considered hubs. The number of connections established by these residues decreases in the open SBDS with respect to the closed structure. This is evident from Figure 8, where the ∆Degree, which is the difference between the degree of a hub in the open WT and the degree of a hub in the closed WT, is negative for most residues. It should be noted that among the identified hubs there are none of the residues mutated in the present work, so their structural importance cannot be explained by a central role in the PSN of the analyzed structures.

**Figure 9:**
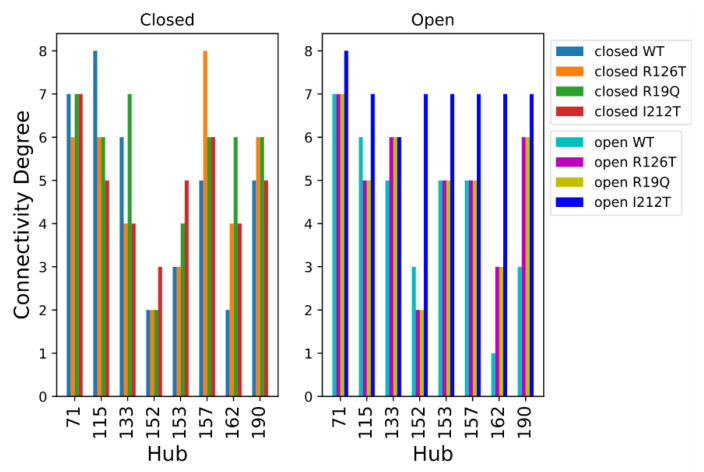
Hub degree comparison of all systems. Residues are enumerated in PDB notation; thus, the correct position can be obtained by adding 2 to the PDB index (e.g., residue R126 is found at index 128).

The analysis of hubs for the PSNs of the wild type and mutated structures is shown in Figure 9, where the residues were considered hubs if they had a connectivity degree higher than 6 in at least one of the eight systems. Looking at the hubs of networks of closed systems, only 4 residues (aa 73, 117, 135, 159) display a connectivity degree higher than 6 in at least one of the four systems. The PSNs of open structures present a higher number of hubs with connectivity degree higher than 6 in at least one of the four systems, namely residues 73, 117, 154, 155, 159, 164 and 192. However, most of the selected hubs (except residue 73) had a high connectivity only in the open I212T PSN, while the connectivity degree was much smaller in other cases. Comparing these results obtained for closed and open systems, we can say that residue 73 is an important hub in all the considered networks and that the open I212T conformation has a higher connectivity with respect to the other open structures.

### 2.6. The Role of the C-Terminal Domain

**Figure 10:**
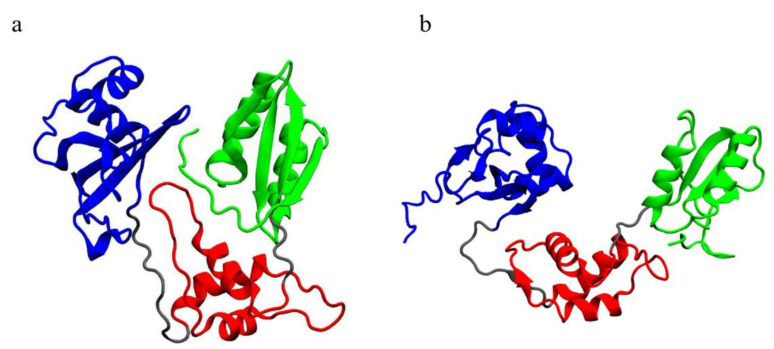
Visualization of the equilibrated trajectories: (**a**) closed WT and (**b**) open WT. Different colors indicate different domains: blue, domain I; red, domain II; green, domain III; grey, linkers. The C-terminal domain is clearly in contact with both domains I and II in the closed conformation.

Open mutated systems display lower mobility in their C-terminal domain, with respect to the WT (Figure 3b). A visual inspection (Figure 10) points out that the C-terminal domain is crucial for the interaction between different domains of SBDS, especially in its closed form, although displaying different behaviors in the investigated systems. Within our study, we observed that the C-terminal domain comes in contact with both domains I and II.

**Figure 11:**
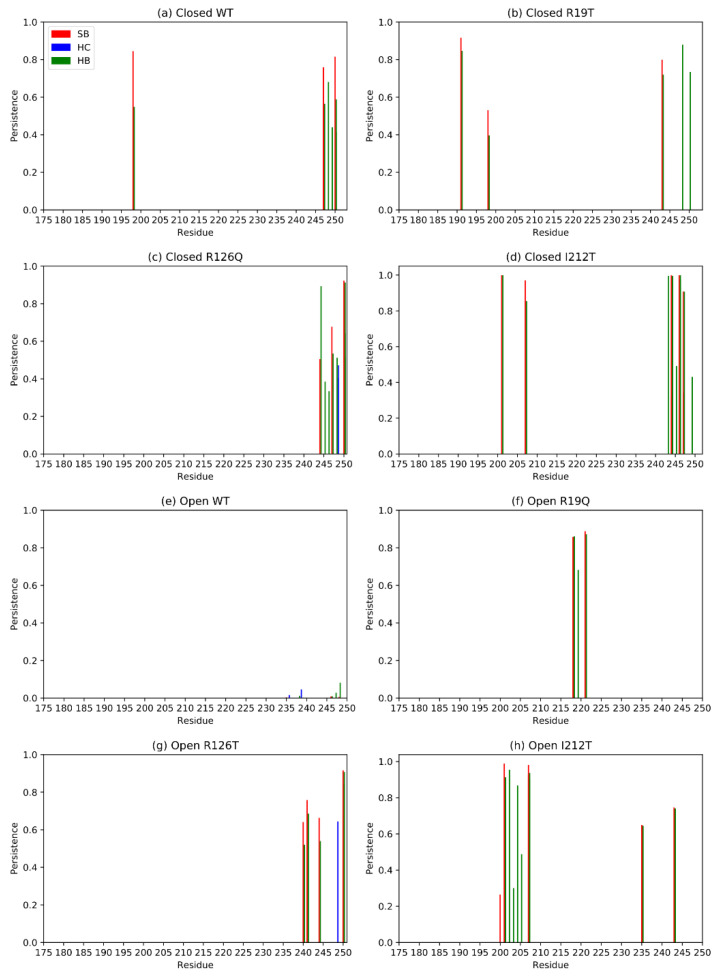
Principal interactions between residues belonging to domains I and III for each system. SB stands for salt bridge, HC for hydrophobic interactions and HB for hydrogen bonds.

Distant domains, whose relative motion is essential for the functionality of the protein, may interact through non-covalent bonds. These interactions contribute to determining the compactness of the structure, and they are often represented by salt bridges [25]. With this in mind, we analyzed the interactions between domain I and domain III of SBDS in the different systems. Exploiting Pyinteraph, we retrieved the persistence and the nature of each non-covalent bond between residues belonging to domains I and III. Figure 11 reports only the values of persistence that exceed the specific threshold p_crit_ for each interaction type for domain III. Panels a, b, c and d report the persistence of interactions between residues of domain III and those of domain I for closed systems (not displayed here). The plots point out the crucial importance of the C-term for this inter-domain interaction, as already hypothesized by visual inspection. In the case of open systems, the open WT has no interactions above the threshold, hence in this case domains I and domain III do not interact directly (Figure 11e), as confirmed also by visual inspection (Figure 10). The C-terminal contribution does not seem crucial for the open systems, as can be noted by inspection of Figure 11f–h, but for the open R126T. Notably, however, open mutants tend to establish more inter-domain interactions with respect to the WT, probably contributing to their overall stability and resemblance to an intermediate state between the open and closed WT conformation. Overall, salt bridges and hydrogen bonds are more present than hydrophobic interactions, as expected for residues on protein surfaces.

## 3. Discussion

Our results indicate that the point mutations herein investigated might affect the SBDS conformational landscape and offer insights into the allosteric connections occurring between the three domains of the protein. More specifically, the *RMSD* analysis of the closed conformations clearly suggests that domain III is strictly interconnected with the other two domains, since the herein-explored mutations on domains I (R19Q) and II (R126T) both strongly affect the dynamics of domain III. Published experimental evidence [23] indicates that both these mutations can strongly affect the so-called “second binding event” with EFL1. Worthy of note is that the replacement of R126 (R126T) proved to affect also the first binding stage. Building on these pieces of evidence, we can herein postulate that the conformation of domain III might play a role, not only in the first event (as reported in reference [23]) but also in the second one, required for an effective SBDS-EFL1 interaction. Domain II, on the contrary, seems to be not affected by instabilities introduced on other domains, at least as far as the hereby-investigated mutations are concerned. Other analyses support the importance of domain III also in the conformational behavior of the open states. The computed *RMSF* clearly showed that the considered mutations in the open forms strongly stabilize this domain. This is especially evident if we focus our attention on the C-terminal of the protein (residues 225–252). As emerged from our Pyinteraph investigation, such an effect is the consequence of the protein closure resulting from an increase of the interactions occurring between domain I and domain III. Importantly, this is observed irrespective of the considered mutation. In other words, the picture emerging from these data suggests that when involved in strong interactions with domain I, domain III might not be available for other interactions; including the interaction with EFL1. Our data also point out that a dynamical equilibrium between the two distinct conformations (open and closed) is crucial to ensure a proper SBDS function, in full agreement with our previous observations [23,24]. Importantly, all the investigated mutants, irrespective of the considered starting conformation (open or closed), tend to assume an intermediate state, as evident from the computed ∆σ. Remarkably, and in full agreement with the experimental observations, the I212T mutant, differently from R19Q and R126T both affecting only the binding effect, has been reported to severely disrupt EFL1 binding [23,26] and has displayed the highest propensity to reach an intermediate state regardless of the starting conformation (whether open or closed). Finally, our data put forward residue 73 as an important hub for SBDS interdomain communication and, thus, strongly indicate that any mutation on this site would severely affect either the conformational stability of the protein or the dynamical equilibrium between the open and closed states. Although many steps in the onset of Shwachman–Diamond Syndrome still need further clarification, our proposed comparative Molecular Dynamics assessment of different SBDS mutants may set a standard protocol for further investigations: the trajectories obtained starting from the open and closed forms (or even intermediate states) provide useful insight to interpret a large amount of experimental data. In addition, they also suggest mutations that have not yet been explored and may strongly affect the delicate dynamical equilibrium between the two states. This evidence cannot be obtained by merely looking at coordinates obtained in crystal structures, if available: dynamical equilibria can be assessed only by fully exploiting the possibilities of MD simulations.

## 4. Methods

### 4.1. Model System Preparation

The coordinates of the WT human SBDS model were extracted from deposited PDB structures resolved by NMR (PDB code: 2KDO [26]). From twenty conformers available, two were chosen as representatives of a “closed” and an “open” conformation, namely conformers number 2 and 5, respectively, and used as the initial configurations for model systems preparation. These models were first prepared by adding the missing hydrogen atoms with the optimal protonation states for the histidine residues at physiological pH. The structures of the human SBDS mutants (R19Q, R126T and I212T) were built using the previously modified WT structure, employing the “psfgen” structure building package available within the Visual Molecular Dynamics (VMD) software suite [27]. Each of the eight systems was then incorporated into a periodic box of TIP3P water molecules [28] extended by 14 Å in all directions from protein atoms, using the “solvate” package of the VMD Tcl interface. All systems were neutralized with a concentration of 150 mM KCl using the VMD’s “autoionize” plugin.

### 4.2. MD Simulations Set Up

All-atom MD simulations were performed using NAMD 2.13 [29], employing the CHARMM36 force field [30]. Possible steric clashes in the initial geometries were removed by employing a minimization and equilibration protocol: the structures were initially minimized for 1000 steps before undergoing a 450-ns-long MD simulation. Periodic boundary conditions were applied in all directions. A cut-off of 12 Å was applied to the Lennard–Jones interactions employing a switching function (switching radius of 10 Å). Electrostatic interactions were treated using the Particle-Mesh-Ewald (PME) method, with a real-space cutoff of 12 Å and a grid spacing of about 1 Å per grid point in each direction. All simulations were performed in an isothermal–isobaric ensemble, at 1 atm and 310 K, with a Nosè–Hoover Langevin barostat and a Langevin thermostat (damping coefficient 1 ps-1). We used a time step of 2 fs, storing the coordinates every 50,000 steps (100 ps). All simulations were performed on the HPC cluster at the University of Trento (Italy) and on the MARCONI100 supercomputer at CINECA, Bologna, Italy.

### 4.3. Assessment of Equilibration (RMSD)

The analysis of trajectories included standard measures, such as the *RMSD*, the *RMSF* and *R_g_*. These analyses were all performed using the MDAnalysis library in Python (Python 3.6.9; MDAnalysis version 0.20.1) [31,32].

The *RMSD* is defined as:

$$RMSD\left(t\right)=\sqrt{\frac{1}{N}\;\overset{N}{\underset{i=1}{\sum }}{\left(r_{i}\left(t\right)-r_{i}^{ref}\right)}^{2}}$$

where $r_{i}\left(t\right)$ are the coordinates of atom *i* at time *t*, *N* is the number of (Cα) atoms and $r_{i}^{ref}$ are the coordinates of atom *i* in the reference structure. In the present work, the reference structure was chosen as the first frame of each simulation. The *RMSD* of Cα atoms from their reference positions were computed after performing a rotational and translational superimposition of the backbone to the reference structure. The *RMSD* was employed to provide an estimate for the equilibration points of each production run: the part of the trajectory following the equilibration point was taken as a production run and all subsequent analyses were performed on the production run.

### 4.4. Root Mean Square Fluctuations (RMSF)

The *RMSF* are defined as:

$$RMSF\left(i\right)=\sqrt{\frac{1}{T}\overset{T}{\underset{t=1}{\sum }}{\left(r_{i}\left(t\right)-r_{i}^{ref}\right)}^{2}}$$

where $r_{i}\left(t\right)$ are the coordinates of atom *i* at time *t*, *T* is the number of frames of the simulation and $r_{i}^{ref}$ are the coordinates of atom *i* in the reference structure. The average structure of each simulation was taken as a reference. In the present work, *RMSF* was computed for each Cα atom of the protein as the representative atom of an amino acidic residue. *RMSF*s were computed after aligning the trajectory to the reference structure to remove translational and rotational artifacts.

### 4.5. Radius of Gyration

The *R_g_* is defined as:

$$R_{g}\left(t\right)=\sqrt{\frac{1}{M}\overset{M}{\underset{i=1}{\sum }}m_{i}(r_{i}\left(t\right)-R)}$$

where *M* is the total mass of the atoms in the protein, *m_i_* is the mass of the *i*-th atom, *r_i_* are its coordinates and ***R*** are the coordinates of the center of mass.

*R_g_* is computed over time to analyze the evolution of the general compactness of the protein structure, and it was here employed as an indicator of the motion of domains towards a more open or closed functional conformation.

### 4.6. Distances and Angles

Distances between domains of the protein were computed as the centers of mass of the domains (considering Cα atoms only). Angles between different protein domains were computed between the centers of the geometry of their Cα atoms.

The angles were computed as:

$$\theta =\arccos\;\left(\frac{BA\;\cdot BC}{\left|BA\right|\left|BC\right|}\right),$$

where *A*, *B* and *C* represent the centers of mass for the *A*, *B* and *C* subunits, respectively.

### 4.7. Analysis of Protein Structure Hinges

To identify the hinge regions of the protein and visualize their principal slowest motions (first and second), the HingeProt program and its web server service were used [33]. For this purpose, the first frame structure file (in PDB format) of each production run was analyzed.

### 4.8. Protein Structure Network Generation and Analysis

The tool used to study the PSN of all the SBDS systems was Pyinteraph [34], a Python library that allows the creation and analysis of non-covalent interactions for a given structure and trajectory.

Briefly, the software calculates the percentage of frames of MD trajectories in which distance and angle constraints peculiar to each non-bonded interaction (electrostatics, H-bonds and hydrophobic interactions) are fulfilled. An exhaustive list of such constraints is detailed in [34].

A persistence threshold p_T_ was calculated according to the size of the largest hydrophobic cluster criterion [35] for each simulated system. These values were used to filter the three respective interaction graphs, before merging them in a single PSN representing the specific simulated system.

In the analysis of PSN, hubs of the network were defined as residues having a connectivity degree (number of non-covalent bonds formed during the simulation) higher than 6 (*k* > 6) in at least one of the considered systems, if not specified otherwise.

Δ*Degree* was computed for the comparison of the connectivity degree of hubs in wild-type systems. It is defined as:

$$\Delta Degree_{i}=k_{i}^{open}-k_{i}^{closed}$$

where *i* is the *i*-th residue considered and *k* is the connectivity degree of such residue in the closed $k_{i}^{closed}$ and open PSN $k_{i}^{open}$.

## 5. Conclusions

The MD data reported here complement the static view resulting from the available NMR structures of SBDS, a protein whose mutations are responsible for a rare ribosomopathy with a wide spectrum of clinical presentations. The picture that emerged from the discussed data put forward domain III of the protein as crucial for the interaction with EFL1, considered essential for a proper ribosome functioning. In particular, our investigation indicates: (i) the presence of a competition between the domain I of the protein and EFL1 for the interaction with the domain III; (ii) the importance of residue 73 for SBDS conformation/function. Hence, the MD approach may prove useful in facilitating the development of therapeutics that, by altering the SBDS conformation, might counteract the effects of the loss-of-function mutations investigated herein. In other words, our study provides a valuable starting point for directing ad hoc experiments aimed at understanding, from a molecular point of view, the SDS pathogenesis and, therefore, developing the first molecular strategy for a focused SDS therapy.

## Figures and Tables

**Table 1:** Equilibration points set for trajectories of different structures.

Structure		Equilibration Point (ns)
closed	WT	300
	R126T	380
	R19Q	350
	I212T	300
open	WT	400
	R126T	320
	R19Q	350
	I212T	300

## Data Availability

All data, including the analyzed MD trajectories, are available upon request.
